# Marriages of Consanguinity

**Published:** 1864-06

**Authors:** 


*Marriages of Consanguinity.*—M. de Criqc-Cassaux, with a view to refute the arguments lately brought forward to prove the danger of marriages amongst relations, quoted at the last sitting of the Academy of Sciences the example of the ancient kings of Persia, who, since the time of Cambyses, had been in the habit of marrying their sisters, and even their daughters, and yet produce a very fine race.

